# Assessment of the mechanical role of cranial sutures in the mammalian skull: Computational biomechanical modelling of the rat skull

**DOI:** 10.1002/jmor.21555

**Published:** 2023-01-30

**Authors:** Alana C. Sharp, Hugo Dutel, Peter J. Watson, Flora Gröning, Nick Crumpton, Michael J. Fagan, Susan E. Evans

**Affiliations:** ^1^ Department of Musculoskeletal and Ageing Sciences, Institute of Life Course and Medical Sciences University of Liverpool Liverpool UK; ^2^ Department of Cell and Developmental Biology University College London London UK; ^3^ Department of Engineering University of Hull Hull UK; ^4^ Faculty of Science, School of Earth Sciences University of Bristol Bristol UK; ^5^ School of Medicine, Medical Sciences and Nutrition University of Aberdeen Aberdeen UK

**Keywords:** cranial sutures, craniofacial, feeding, finite element analysis, multibody dynamic analysis, rodent

## Abstract

Cranial sutures are fibrocellular joints between the skull bones that are progressively replaced with bone throughout ontogeny, facilitating growth and cranial shape change. This transition from soft tissue to bone is reflected in the biomechanical properties of the craniofacial complex. However, the mechanical significance of cranial sutures has only been explored at a few localised areas within the mammalian skull, and as such our understanding of suture function in overall skull biomechanics is still limited. Here, we sought to determine how the overall strain environment is affected by the complex network of cranial sutures in the mammal skull. We combined two computational biomechanical methods, multibody dynamics analysis and finite element analysis, to simulate biting in a rat skull and compared models with and without cranial sutures. Our results show that including complex sutures in the rat model does not substantially change overall strain gradients across the cranium, particularly strain magnitudes in the bones overlying the brain. However, local variations in strain magnitudes and patterns can be observed in areas close to the sutures. These results show that, during feeding, sutures may be more important in some regions than others. Sutures should therefore be included in models that require accurate local strain magnitudes and patterns of cranial strain, particularly if models are developed for analysis of specific regions, such as the temporomandibular joint or zygomatic arch. Our results suggest that, for mammalian skulls, cranial sutures might be more important for allowing brain expansion during growth than redistributing biting loads across the cranium in adults.

## Graphical abstract

- Cranial bones are connected to each other by sutures.
- Using the rat cranium as a model system we aimed to investigate how sutures impact bone strain distribution and magnitude during feeding.
- By combining quantitative dissections and finite element analysis we showed that the presence of sutures does not change the overall cranial bone strain distribution. However, sutures appear to affect the structural behaviour of certain regions of the cranium.

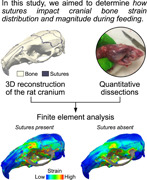

## 1 INTRODUCTION

Cranial sutures are fibrocellular joints between the skull bones and are sites for bone deposition and growth throughout ontogeny (Opperman, 2000). The progressive replacement of suture material with bone facilitates alterations in length, width, and shape of the head as maturation proceeds. The ossification of cranial sutures modifies their biomechanical properties; therefore, suture mechanobiology influences the pattern of mechanical force experienced and transmitted by the craniofacial complex during growth. Mechanical insights into the behaviour of the skull and the influence of cranial sutures have, therefore, proven valuable when studying cranial dysmorphologies, such as craniosynostosis (Moazen et al., 2009; Sharma, 2013).

In adult skulls, sutures are often described as fibrous and relatively immobile joints, yet sutures can be sites of skull mobility, especially in more patent (unfused) contacts like those in reptiles (Smith & Hylander, 1985). Multiple in vitro and in vivo studies have attempted to characterise the mechanical environment of sutures (tensile or compressive) across various species (Behrents et al., 1978; Byron, 2009; Herring & Mucci, 1991; Jaslow & Biewener, 1995; Rafferty & Herring, 1999; Shibazaki et al., 2007; Shibazaki‐Yorozuya et al., 2012). However, although in vitro and in vivo studies are of value in determining local strain environments, it is important to exercise caution when inferring whole‐skull responses to loading (Curtis et al., 2013). Finite element (FE) modelling has shown that cranial sutures can influence strain magnitude and distribution within the skull of mammals, reptiles, and birds (Bright, 2012; Cuff et al., 2015; Curtis et al., 2013; Jones et al., 2017; Kupczik et al., 2007; Moazen et al., 2013). Further studies have described the relationship between loading direction, degree of suture complexity and energy absorption when modelling suture mechanical behaviour, and this introduces an additional layer of complexity when discerning suture function (Jasinoski et al., 2010b; Maloul et al., 2014). Therefore, despite an increasing number of studies, our understanding of overall suture function overall in skull biomechanics is still limited.

FE analysis is an engineering method that has become widely used to test functional hypotheses of complex structures in fields including biomedical sciences, ecology and palaeontology (Rayfield, 2007; Richmond et al., 2005). It can provide insight into the function of structures that cannot otherwise be tested with in vivo and in vitro methods (Panagiotopoulou et al., 2020; Ross et al., 2011; Smith et al., 2021), as well as testing hypothetical morphologies (Dutel et al., 2021; Jones et al., 2017; Sharp & Rich, 2016; Tanner et al., 2008). With advances in microcomputed tomography (µCT) and computing power, more detailed, higher resolution models with more elements are becoming easier to produce and analyse, leading to a more realistic representation of the skull geometry and more accurate results (Bright & Rayfield, 2011; McCurry et al., 2015; Tseng et al., 2011). This has helped to circumvent some limitations inherent to in vivo approaches (e.g., strain gauges) to investigate the mechanical consequences of morphological variation. For example, strain gauges are highly invasive and can alter animal behaviour (Ross et al., 2018), and they can only record in vivo strain where they are located, which can be limited by specimen size and morphology. More bio‐realistic models have the potential to allow the replacement, refinement, and reduction (3Rs) of experiments using animal models in biomedical and veterinary research, and ultimately to build accurate human in silico models. However, many challenges still exist for measuring and representing the material properties of soft tissues such as sutures, ligaments and fascia.

When balancing model complexity with sufficient accuracy for the questions being addressed, soft tissues such as sutures, ligaments and fascia are often excluded. The mechanical role of these soft tissues in overall cranial mechanics therefore remains poorly understood. The influence of these simplifications has been examined in various sensitivity and validation studies (e.g., Curtis et al., 2011; Fitton et al., 2012, 2015; Gröning et al., 2012, 2013; McCormack et al., 2017; Strait et al., 2005). In studies of cranial biomechanics, the role of cranial sutures has received a lot of attention, and in most studies their inclusion in FE models is found to increase the magnitude and change the orientation and distribution of strains in parts of the cranium (Bright, 2012; Curtis et al., 2013; Jones et al., 2017; Moazen et al., 2009, 2013). Strains within sutures will inevitably reach magnitudes greater than the adjacent bone strains, because of the sutures’ lower stiffness, with strains in the bone being reoriented and/or damped compared to models without sutures (Herring & Teng, 2000; Jaslow & Biewener, 1995; Rafferty & Herring, 1999). Sutures may also help to dissipate stress more evenly over the skull in reptiles and this could help to maintain bone growth and health (Curtis et al., 2013; Dutel et al., 2021; Jones et al., 2017; Moazen et al., 2009).

However, studies that include sutures in FE models have not necessarily increased the accuracy of the model when compared to in vivo and in vitro experimental data (Bright, 2012; Cuff et al., 2015; Wang & Dechow, 2016; Wang et al., 2010). This could be due to multiple factors, including the material properties used in the model, simplification of the suture network and the structure of the sutures themselves, or limitations and effects of the experimental approaches (Ross et al., 2018). Sutures can assume a variety of structural types, from simple “flat” sutures to complex interdigitated sutures. Butt‐ended, or end‐to‐end, vertical wall, flat and plane sutures are the simplest and have articulating surfaces orientated approximately perpendicular to the surface of the bone, but more complex interdigitated sutures have convoluted bony processes that vary in size and frequency, and change depending on location and age of the animal (Curtis et al., 2014; Jones et al., 2009). Most studies do not model the complexity of the sutures, and only model a limited set (Bright, 2012; Moazen et al., 2013; Wang et al., 2010). However, the structure of sutures does have an effect on local strain patterns and magnitude experienced in the bone (Curtis et al., 2013; Dzialo et al., 2014; Jasinoski & Reddy, 2012; Jasinoski et al., 2010a; 2010b; Maloul et al., 2014; Rafferty & Herring, 1999; Wang & Dechow, 2016), but no studies have yet looked at wider, whole skull effects of these structural changes in mammals.

Considering this complexity, our aim was to build an anatomically detailed biomechanical model of the rat skull to evaluate the mechanical role of the sutures on cranial strain during biting. The albino rat (*Rattus norvegicus*) was chosen because it is common throughout laboratories around the world and is an important model species for research in biomechanics, biomedical and behavioural sciences (Meakin et al., 2014; Shibazaki et al., 2007; Shibazaki‐Yorozuya et al., 2012). Rats have also been an important species in understanding the relationships between diet, ecology, and evolution within rodents (Cox et al., 2011, 2012; Ginot et al., 2018). In the rat, cranial sutures remain patent throughout life, except the posterior part of the interfrontal suture, which undergoes fusion by 21 days after birth (Moss, 1954, 1957). This makes the rat a good model for biomechanical studies on suture function and suture mobility in adult phenotypes. Moreover, as its overall cranial architecture differs strongly from that of other well‐studied skulls, such as those of pigs (Bright, 2012) and primates (e.g., Dumont et al., 2011; Fitton et al., 2012), the rat has the potential to contribute to a broader overall understanding of the influence of feeding forces on cranial design in mammals.

We hypothesise that including cranial sutures in our model will impact cranial bone strains. Based on previous results obtained in lizards (Curtis et al., 2013; Dutel et al., 2021; Jones et al., 2017; Moazen et al., 2009), we expect that including sutures in the model will increase the overall cranial bone strain magnitudes and decrease strain gradients across the cranium, since the lower elastic modulus of sutures compared to bone might allow for loads to be efficiently dissipated across the cranium as in lizards. This would have implications for model accuracy when choosing which soft tissue structures to include and may even impact comparative studies if models change relative to each other due to differences in species morphology, suture patency, and material properties.

## 2 METHODS

### 2.1 In vivo bite force

In vivo bite force was measured at the incisors using a piezoelectric isometric Kistler force transducer (9311B; range: ±5000 N) (Aguirre et al., 2002; Herrel et al., 1999). Force magnitudes of a series of five voluntary bites were measured at the incisors on one adult male domestic rat (*Rattus norvegicus f. domestica*). As muscle isometric force relates to muscle fibre length (for review see Miller, 2018), muscle force and bite force are expected to vary with jaw gape. To avoid excessive muscle fibre stretch, we therefore kept the bite plates of the transducer at minimal distance (about 3 mm). The maximal bite force magnitude measured was retained for comparison with a multibody dynamics analysis (MDA) model. All experimental procedures were performed at the Museum national d'Histoire naturelle, Paris, France, under ethical approval in accordance with French law.

### 2.2 Dissection and muscle morphology

The male rat specimen was euthanised by an intramuscular injection of pentobarbital. The head muscles were dissected one by one from the defrosted cadaver, with each being photographed in situ before removal to ensure correct orientation. Muscles were immediately weighed (wet weight) and pennation angle was measured where applicable. For muscles that were not highly pennate, or parallel fibred, pennation angle was recorded zero. Muscles were placed into a 20% aqueous solution of nitric acid for 4–6 h to separate the individual muscle fibres. Nitric acid was replaced by a 50% aqueous solution of glycerol to stop the digestion, and 10‐20 muscles fibres were randomly selected and photographed. The length of each fibre was then measured using the software Fiji (Schindelin et al., 2012) to calculate the average fibre length of each muscle.

The physiological cross‐section area (PCSA, in cm^2^) of each muscle was calculated using the following equation (Sacks & Roy, 1982):

$$\mathrm{PSCA}=\;\frac{\mathrm{mass}∙\cos(\alpha )}{\mathrm{fl}∙\rho }$$

where mass is the muscle mass (in g), α the mean pennation angle of the muscle fibres (in degree), fl the mean resting fibre length (in cm), and ρ the muscle fibre density of 1.06 g cm^−3^ (Méndez, 1960). Maximal isometric force was then calculated by multiplying each muscle PCSA with a constant value of muscle stress of 25 N cm^−2^.

### 2.3 Tomography and segmentation

**Figure 1:**
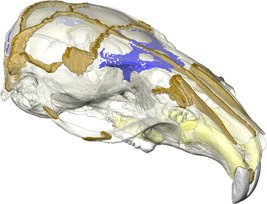
Finite element model of the rat cranium showing the sutures (brown), trabecular bone areas (blue), PDL (yellow), teeth (white) and bone (transparent).

Before dissection, the head of the rat specimen was scanned at the University of Hull, UK, using an X‐Tek HMX160 μCT system (X‐Tek Systems Ltd.) with a voxel size of 0.031 mm in each direction. After reconstruction, the image stacks were saved in *.tiff file format and imported to Avizo 9.5.0 (FEI Visualisation Sciences Group) for segmentation. Six structures were manually segmented: cortical bone, trabecular bone, sutures, teeth (combined enamel and dentine), dental pulp cavity, and the periodontal ligament (PDL; Figure 1). The sutures were segmented as accurately as possible to represent their complex interdigitated structure and were 0.2 mm thick minimum, except where sutures were completely fused (e.g., interfrontal). Similarly, the PDL was included for each tooth and modelled by a thin (0.2 mm) layer between the alveoli and tooth roots, because there is strong evidence that inclusion of PDL in FE models also modifies cranial strain (Gröning et al., 2011; McCormack et al., 2017). The trabecular bone was modelled as a solid structure nested within the cortical bone volume.

### 2.4 Multibody dynamics model

An MDA model was constructed in ADAMS v.2021.0.1 (MSC Software Corp.) with two rigid bodies, the cranium (fixed part) and mandible (moveable part). To allow a realistic range of motion, the temporomandibular joint (TMJ) was modelled through contact analysis which enabled movement of the jaw in all degrees of freedom (DOF). Mass and inertial properties of the mandible were calculated within ADAMs based on the mesh volume and a standard tissue density of 1.05 g cm^−3^ (Sellers & Crompton, 2004). Muscles were discretized into a series of strands connecting their origin and insertion sites. When required, muscles were wrapped around the bone to represent the orientation of their line of action as accurately as possible. The maximum isometric muscle force was then divided by the number of strands representing the muscle, and this force assigned to each strand of the muscle.

For the purpose of the present study (e.g., to test the influence of sutures on cranial bone strain under maximal load), only maximal bite force was simulated at the incisor and the premolar (most anterior along the tooth row). The detailed dynamic aspects of this model will be presented in a future study and follow the approach by Watson et al. (2014). To estimate the accuracy of our model, we then compared the maximal bite force predicted by our model to the maximum recorded in vivo bite force measured on the same specimen.

### 2.5 FE analysis

The FE mesh was generated in Avizo and consisted of 7,995,364 4‐node tetrahedral elements. Adaptive meshing was used to limit the number of elements, while being sufficiently fine to represent small and thin structures such as the sutures and the PDL. The mesh was then converted to.txt format using a custom‐made R (R core Team, 2014) script and imported to ANSYS v.17.2 (Swanson Analysis Systems), where the linear 4‐node tetrahedral elements were converted into higher‐order 10‐node tetrahedral elements (ANSYS SOLID187).

All materials were idealised as homogeneous, linear elastic isotropic materials. The Young's modulus and Poisson's ratio of each material is based on published values obtained from nano‐indentation measurements. Cortical bone was assigned a Young's modulus (E) value of 19,920 MPa and a Poisson's ratio (ν) of 0.3 (Cox et al., 2012), and the trabecular bone was modelled with E = 56 MPa and ν = 0.3 (Herrel et al., 1999); the PDL and sutures were modelled with E = 50 MPa and ν = 0.49 (Rees and Jacobsen, 1997), and E = 20 MPa and ν = 0.49, respectively. As this study was not concerned with the strains in the teeth, the dentine and enamel were modelled as a single structure and assigned the material properties of enamel (E = 62,370 MPa, ν = 0.33) (Cox et al., 2012), whereas the pulp was modelled with E = 2 MPa and ν = 0.45 (Benazzi et al., 2016).

Muscle forces calculated in ADAMS for each bite location were imported to ANSYS with the force in each muscle strand applied as a nodal load at the strand origin. The path of muscle bundles wrapping over the cranial vault (lateral, medial and posterior temporalis) was modelled in ADAMS by a series of cable actuators connected to each other, a method that has been previously used to model complex muscle paths (e.g., Gröning et al., 2013; Watson et al., 2014). The configuration of wrapped muscle forces was replicated in ANSYS using spring elements (ANSYS LINK180) with tension‐only capabilities. To avoid large displacements, the DOF of the nodes connecting each link element along the wrapped muscle path were coupled with the initial node of the path located on the surface of the cranium. Muscle force extracted from the MDA model was applied to the most distal node of the muscle path. The FE mesh was then constrained as follows: one node was constrained on each incisor in the vertical direction to simulate bilateral biting, and one node at the first left molar was constrained in the vertical direction to simulate posterior unilateral biting. The TMJ was constrained at one node in all three directions at the balancing side, and in the anterior‐posterior and dorsal‐ventral directions on the working side. This set of constraints aimed at avoiding over‐constraining the model and to prevent large strain artefacts.

To assess the influence of sutures on cranial bone strain, two FE analyses were run for each loading case: (1) one on the model with the suture network representing the actual morphology of the specimen; and one on (2) an altered morphology where all the sutures were fused, by assigning the material properties of cortical bone to the sutures.

Element strain results were then exported from ANSYS and converted into *.vtk* files to be visualised in the open‐source software Paraview (Ahrens et al., 2005; www.paraview.org). In addition to contour plots of the principal strains, the |ε_1_:ε_3_| ratio for each element was calculated to determine the dominant principal strain for each element which was then mapped onto the mesh. Difference plots were also generated to visualise the absolute and relative differences in strain magnitude between the model with and without sutures. Principal strain magnitudes for the entire bones were calculated by averaging strain magnitudes of the nodes on their surface. Postprocessing of the ANSYS results to generate.*vtk* files and quantitative data analyses were performed in the software R (R core Team, 2014).

In vivo strain gauge measurements were unavailable for the specimen used in our study. Instead, we have made comparisons with strain gauge measurements conducted by Shibazaki et al., 2007; Shibazaki‐Yorozuya et al., 2012) on 70‐day‐old rats (a maturity equivalent to our specimen). To compare our models against the in vivo data, strain was output at locations on the model that correspond to the strain gauge locations used by Shibazaki et al. (2007) and Shibazaki‐Yorozuya et al. (2012), namely: (1) interfrontal suture (IFS); (2) sagittal suture (SGS); and (3) parietal bone (PB). The strain at each location was averaged from an area of 1 mm^2^, about the size of the rosette strain gauge, at the same position and alignment as Shibazaki's channel 2 gauges.

## 3 RESULTS

### 3.1 Multibody dynamics analysis

The MDA model predicted a maximal bite force at the incisor of 30.58 N, which was close to the maximal in vivo bite force measured on the same specimen (32.60 N) at this location. The FEA predicted a slightly lower bite force at the incisor (28.42 N), probably due to approximations in the position of the muscle forces imported from ADAMS and in the location of the constraints applied to the mesh.

### 3.2 FE analysis

**Figure 2:**
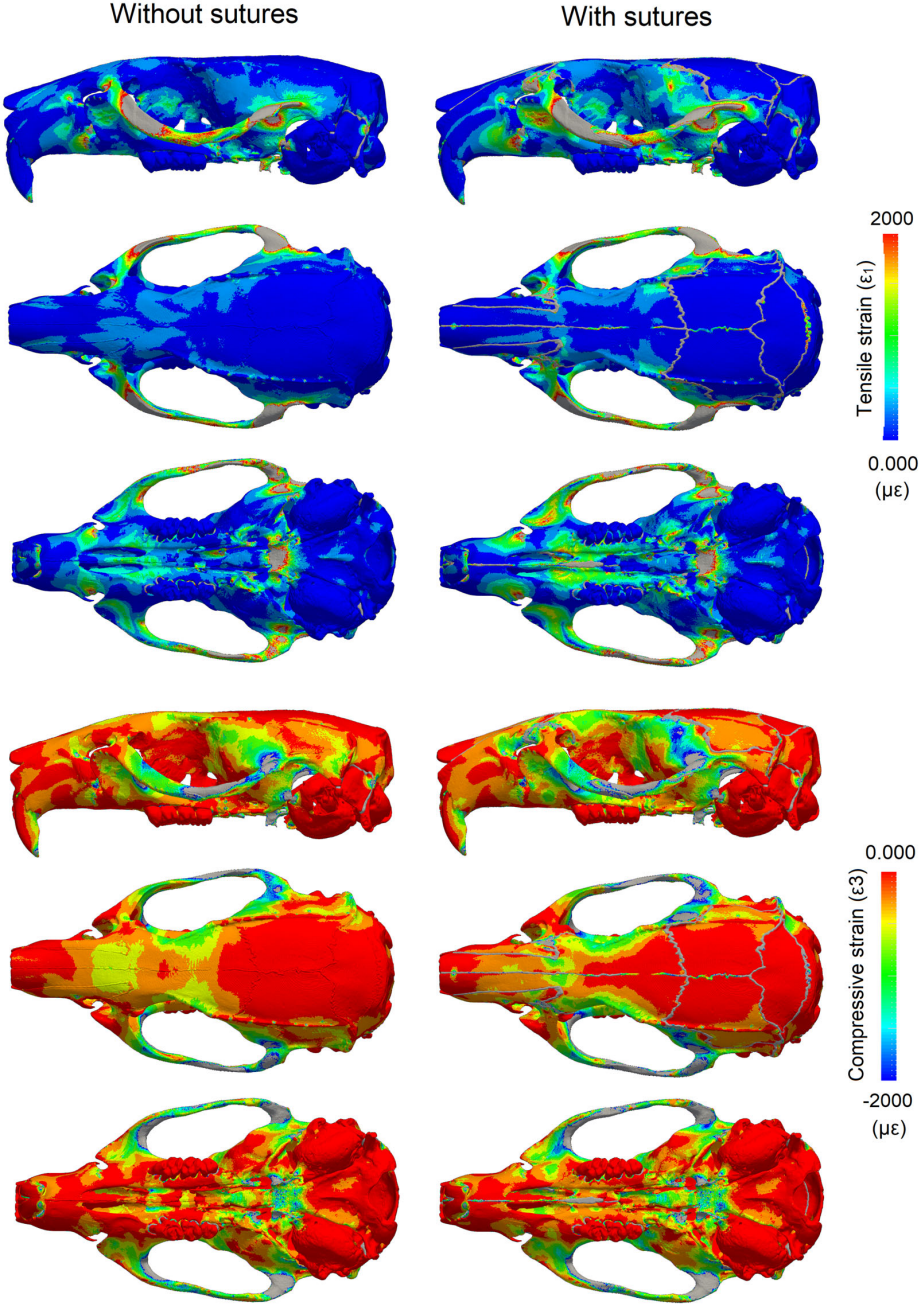
Strain pattern in the rat cranium showing the impact of patent sutures during incisor biting. First (ε1) and third (ε3) principal strain calculated during bilateral incisor biting with and without sutures. Strain magnitude is in microstrain (με); areas in grey correspond to out‐of‐range strain values.

**Figure 3:**
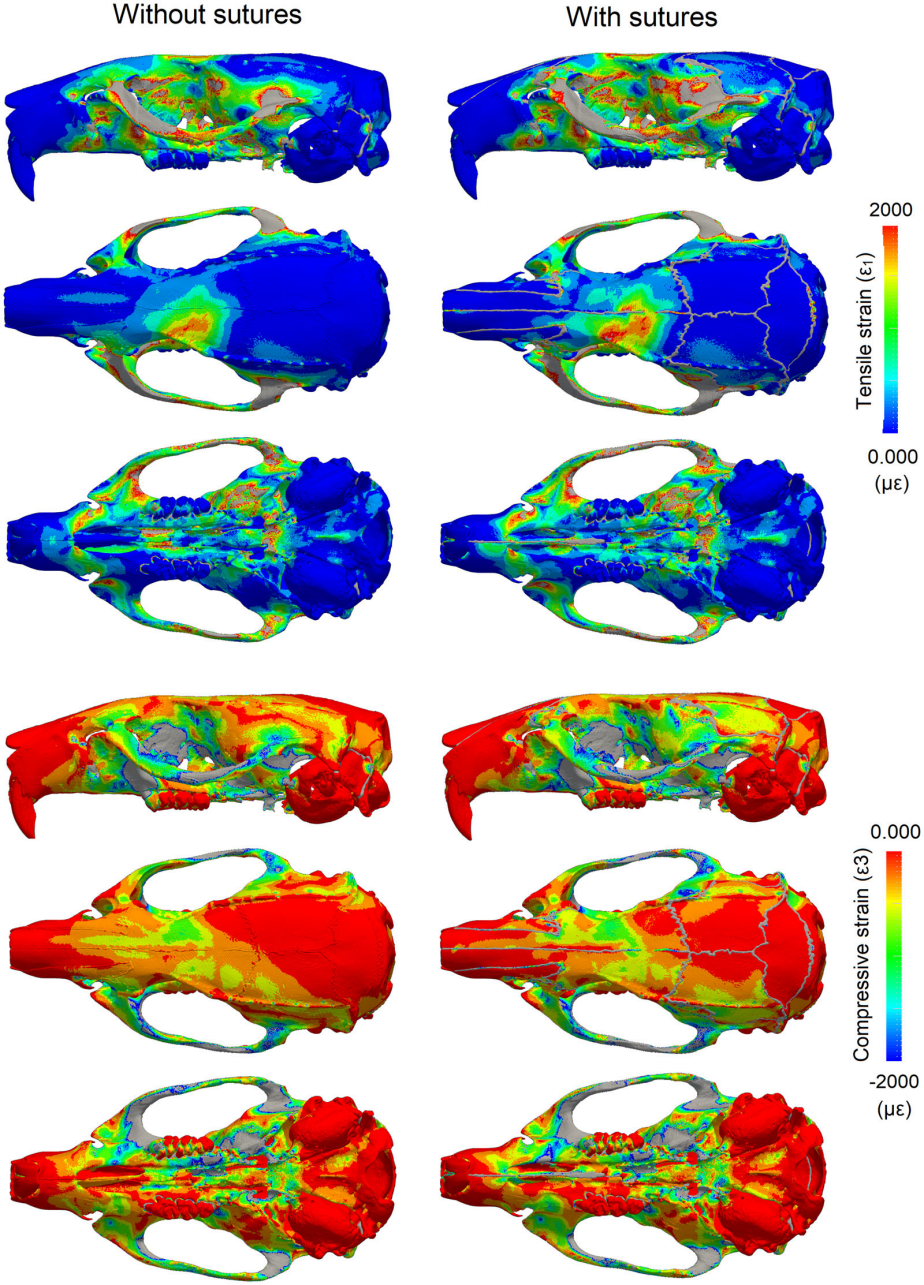
Strain pattern in the rat cranium showing the impact of patent sutures during molar biting. First (ε1) and third (ε3) principal strain calculated during unilateral molar biting with and without sutures. Strain magnitude is in microstrain (με); areas in grey correspond to out‐of‐range strain values.

**Figure 4:**
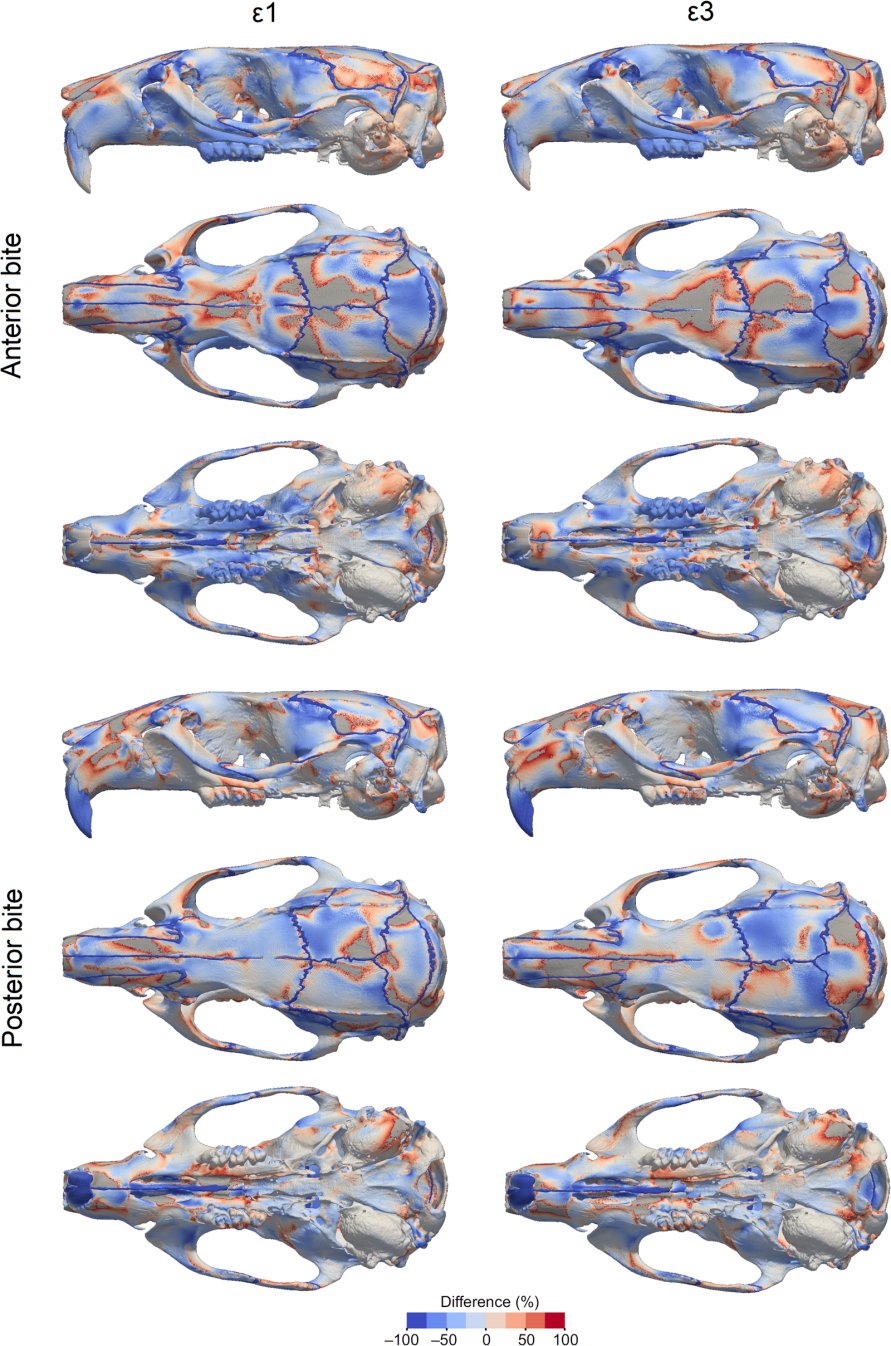
Relative difference in principal strain between models with and without sutures. Negative values (cold colours) correspond to higher strain when sutures are present, while positive values (warm colours) correspond to higher strain when sutures are absent/fused. Areas in grey correspond to out‐of‐range strain values.

Contour plots were produced for the 1st (ε_1_, most tensile) and 3rd (ε_3_, most compressive) principal strains (Figures 2 and 3), as well as difference plots comparing the two models (Figure 4). For all analyses, strain was consistently higher on the working side during unilateral molar biting. The zygomatic arch experienced the highest strain, with tension being dominant at both ends and compression being dominant in the centre of the arch on the dorsal surface. Other areas of relatively high tensile strain included the attachment site for the anterior medial temporalis muscles on the lateral surface of the skull, which also recorded higher strain during molar biting compared to incisor biting. Areas of high compressive strain included the sphenoid bone, where the strain was also much higher during molar biting compared to incisor biting.

When comparing models, the inclusion of sutures made the most notable changes to local strain distribution and magnitude; however, more broadly, overall strain magnitudes and gradients do not substantially change with and without sutures. The model with sutures had higher strain magnitudes compared to the model with no sutures at the strain gauge sites (Table 1), and this varied depending on whether strain was measured near a suture (interfrontal suture or sagittal suture) or further away from a suture (the parietal bone). This varying pattern of strain can also be observed in the contour plots: some areas of bone further from a suture experienced lower strain in the model with sutures compared to the model without sutures, particularly over the dorsal cranium during incisor biting (Figure 2), suggesting some broader, nonlocal effects of modelling sutures. During incisor biting, tensile strain over the skull roof was more symmetrical with sutures than without, and there was an increase in tensile strain over the palate. Also during incisor biting, compressive strain was increased at the nasofrontal suture at the anterior root of the zygomatic arch and decreased at the interfrontal region in the model with sutures compared to the model without. For both tensile and compressive strains, the presence of sutures tended to shift strain more posteriorly in contrast to the model without sutures (Figure 4). For unilateral molar biting, tensile strain increased at the squamous temporal bone, and compressive strain over the temporal region (Figures 3 and 4).

**Table 1:** Strain magnitudes (µɛ) predicted by the models (with and without sutures).

	No sutures	With sutures
Interfrontal suture (IFS)		
Max principal strain (incisor)	60.93 ± 13.44	66.50 ± 9.62
Min principal strain (incisors)	−142.75 ± 25	−37.73 ± 10.1
Max principal strain (molar)	465.78 ± 54.29	625.89 ± 64.65
Min principal strain (molar)	−202.56 ± 18.52	−238.63 ± 28.23
Sagittal suture (SGS)		
Max principal strain (incisors)	33.70 ± 28.8	373.55 ± 1451
Min principal strain (incisors)	−30.80 ± 52.78	−1059.2 ± 4059
Max principal strain (molar)	45.20 ± 131.43	2002.66 ± 3682
Min principal strain (molar)	−45.76 ± 103.66	−1766.31 ± 4173
Parietal bone (PB)		
Max principal strain (incisors)	11.22 ± 2.26	14.82 ± 3.68
Min principal strain (incisors)	−24.73 ± 2.84	−30.23 ± 6.0
Max principal strain (molar)	68.65 ± 14.5	97.89 ± 16.18
Min prin±cipal strain (molar)	−157.53 ± 19.54	−130.20 ± 19.5

*Note*: The mean and standard deviation of maximum principal (tensile) and minimum principal (compressive) strains are given for both incisor biting and molar biting. Note, results from areas that cross over sutures (IFS and SGS) contain both suture and bone material in the model with sutures, so standard deviations are high. *Tensile strains are expressed at positive values and compressive strains as negative values.

**Figure 5:**
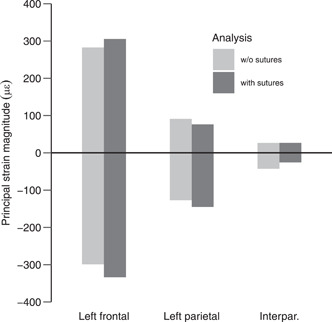
First (positive) and third (negative) principal strain magnitudes on the entire surface of the left frontal, parietal and interparietal bones. Strain magnitudes were averaged for anterior and posterior bites.

Strain recorded in vivo from three locations (Shibazaki et al., 2007; Shibazaki‐Yorozuya et al., 2012)—IFS, SGS, and PB—was compared with strain magnitudes obtained from the models (Table 1 and Figure 5). A general trend in the strain recorded at these locations was observed in both the in vivo data and our models: the PB had the lowest strain, the SGS had the highest, and the IFS had intermediate levels.

## 4 DISCUSSION

Even with the increasing number of studies including cranial sutures, our understanding of their mechanical function and their role in skull biomechanics is still incomplete. Sutures are rarely included in FE models due to their complex three‐dimensional anatomy despite growing evidence of their importance in certain taxa (Curtis et al., 2013; Jones et al., 2017; Markey et al., 2006; Moazen et al., 2009; Rafferty & Herring, 1999). However, for a more complete understanding of skull mechanics it is important to understand the significance of sutures, and other soft tissues, for any particular analysis. Here, a combination of MDA and FEA was used to test the mechanical role of sutures in an anatomically accurate cranial model of a rat.

Previous FEA studies on mammals and reptiles have shown an increase in the magnitude and changes in the local patterns of cranial strain when sutures are modelled (Bright, 2012; Curtis et al., 2013; Jones et al., 2017; Moazen et al., 2009, 2013). In some cases, sutures reduce strain locally, but increase strain in other areas, showing that the changes in strain magnitude are not uniform across the entire skull. Our results from a rat model with an accurate network of cranial sutures, agree with these FEA studies as we see local strain variations between the models (Figure 4). Our results are also in agreement with many experimental studies showing that sutures are generally locations of increased strain compared to the surrounding bone (Table 1). As expected, strain was increased in the suture elements, because of their lower Young's modulus. However, strain decreased in some bone elements directly surrounding the sutures (Table 1), and increased in other areas, including around the nasofrontal suture and the squamous suture (Figure 2).

When compared to experimental measurements of strain in rat crania (Shibazaki et al., 2007; Shibazaki‐Yorozuya et al., 2012), we found that the model without sutures had lower strain than the experimental results and was not able to accurately represent the higher strain at sutural sites (Shibazaki et al., 2007; Shibazaki‐Yorozuya et al., 2012). The sagittal suture is situated between the parietal bones, and during biting the temporalis muscles exert a lateral and downward pull on the parietal bones, causing tension at this suture, illustrated in both our model and the in vivo results (892 ± 485 µɛ) as having the highest recorded strain (Table 1). At the interfrontal suture, which is partially fused at the posterior portion, the strain was predominantly compressive. This is likely due to bending upward of the rostrum of the rat during incisor biting, and is possibly linked to fusion of the suture at this location (Herring, 2008). Strain at the parietal bone site did not change considerably between the models with and without sutures, and was the lowest of the three sites (Table 1), as in the experimental results. In general, sites with sutures (interfrontal and sagittal) had higher strain than sites with no sutures (parietal bone) in both the experimental data and in our model with sutures, but this was not observed in our no‐suture model (Table 1), highlighting how the inclusion of sutures in the model produces results that more closely resemble in vivo data than when sutures are excluded.

It has been argued that sutures are less important in mammals than in species with more open sutures like reptiles (Wang et al., 2010). However, in FEA studies on mammals, only a limited number of simplified sutures have been modelled in any given species (Bright, 2012; Wang & Dechow, 2016; Wang et al., 2010). This does not accurately represent the entire suture network and possible suture interaction, nor the potential importance of suture complexity (White et al., 2021). The morphology of sutures has also been linked to their local strain environment (Cao et al., 2019; Dzialo et al., 2014; Herring, 2008; Liu et al., 2017; Moss, 1954; Rafferty & Herring, 1999). Complex interdigitated sutures are associated with areas under compression (Herring, 2008). Sutures that run transversely, including the lambdoid and coronal sutures, are highly interdigitated in the rat and experience compressive strains perpendicular to the suture (Shibazaki et al., 2007; Shibazaki‐Yorozuya et al., 2012). Conversely, sutures with compressive strains parallel to the suture, such as the frontal and nasal sutures, tend to be deep, straight, and noninterdigitating (Figure 1). Areas under tension in our rat model, particularly during molar biting (e.g., the squamous suture), have broad, flat simple sutures, whereas areas that experience both compression and tension (e.g., the nasofrontal suture at the anterior root of the zygomatic arch), particularly during incisor biting, have long and broad interdigitating fingers. These observations support similar findings reported for local strain environments in primates and Hominins (Dzialo et al., 2014; Wang & Dechow, 2016; Wang et al., 2012). The model without sutures does not display these local strain patterns, and instead effectively acts as a simple beam under sagittal bending during incisor biting. Therefore, we argue that models without sutures cannot accurately estimate the complex strain pattern in mammal skulls with patent sutures like the rat, and that such models that do not include sutures need to be treated with caution especially when making comparisons across species.

However, the inclusion of sutures in the rat model does not substantially change overall strain gradients across the cranium and strain magnitudes in the bones overlying the brain, the frontal, parietal and the supraoccipital (Figure 5). This result contrasts with previous findings on lizards (Dutel et al., 2021; Jones et al., 2017; Moazen et al., 2009) where the inclusion of the sutures markedly increases strain magnitudes in the calvarial bones. In these reptiles, the parietal not only serves as attachment area for the jaw adductors but also resists the loads transferred, by the sutures, from the bite point to the back of the cranium. Despite differences in the architecture of the cranium between rat, rabbit (Watson et al. 2021), and primates (Ross et al., 2011), bone strain magnitude in the calvarial bones of these mammals is always lower, and bone strain distribution is more heterogenous than in lizards. With respect to our present model, this pattern is irrespective of the presence/absence of sutures. Compared with lizards, cranial sutures between the calvarial bones in the rat might hence be more important for allowing brain expansion during growth than redistributing biting loads across the cranium in adults. Our results suggest that calvaria bones in mammals play little role in resisting the feeding loads but rather protect the brain and serve as attachment area for the cranial muscles.

A limitation of this study, and of most studies that model sutures or other soft tissues, is that homogeneous, isotropic material properties were assumed within each of the materials modelled, without considering the complex fibres within the tissues. For example, modelling the PDL as a layer of solid material with constant thickness and linear elastic properties is an approximation that does not take into account the non‐linear properties and the complex morphology of the PDL (McCormack et al., 2017). In our FE model the complete suture network has been modelled in an anatomically accurate way, but assuming homogeneous, isotropic material properties. This may affect the magnitude and direction of strain.

## 5 CONCLUSION

Including anatomically accurate sutures in an FE model of the rat cranium altered local strain magnitudes and patterns (Figure 4). These results show that, during feeding, sutures may be more important in some regions than others. Sutures should therefore be included in models that require accurate strain magnitudes and patterns of cranial strain, particularly if models are developed for analysis of specific regions, such as the TMJ or zygomatic arch. However, overall strain gradients across the cranium and strain magnitudes in the bones overlying the brain, did not change substantially between our suture and non‐suture models, highlighting that in the rat, cranial sutures between the calvarial bones might be more important for allowing brain expansion during growth than redistributing biting loads across the cranium in adults.

In comparative studies, sutures may contribute to differences in strain patterns among taxa that have different degrees of suture fusion or complexity. For example, within mammals, rats have more patent sutures compared to primates, which may be related to overall cranial shape or function. Failure to model these taxon specific differences could affect ecological or functional conclusions, particularly in taxa specialised for enhanced craniofacial use, where suture complexity tends to be greater, such as eating hard foods (Byron, 2009; Byron et al., 2018), antler‐sparring/head‐butting (Farke, 2008; Jaslow & Biewener, 1995), or fossorial lifestyles (Buezas et al., 2017). Therefore, comparisons using models that do not include sutures, need to be undertaken with caution if drawing ecological conclusions across taxa with widely varying cranial shape or functions. These examples, and our results, indicate that cranial sutures may have a more complex and varied role in distributing bone strain in the adult mammalian skull, with varying functions depending on ecology and ontogeny, highlighting that more studies need to include sutures for this to be fully understood.

## AUTHOR CONTRIBUTIONS

**Alana C. Sharp, Hugo Dutel, Flora Gröning, Peter J. Watson, Nick Crumpton**: designed and performed the research, digitised specimens, analysed the data and wrote the paper. **Flora Gröning, Susan E. Evans, Michael J. Fagan, Peter J. Watson, Nick Crumpton**: helped with the interpretation of the data and reviewed drafts of the paper. **Alana C. Sharp**: wrote the original draft. **Susan E. Evans, Michael J. Fagan**: acquired the funding. All authors contributed to designing the experiments and approved the final draft.

## CONFLICT OF INTEREST STATEMENT

The authors declare no conflicts of interest.

## Data Availability

The data that support the findings of this study are openly available at https://figshare.com/projects/Sharp_et_al_2023_J_Morphol_/157557. These data include anatomical data used as input for the model, and the.vtk files containing the 3D mesh and principle strain results that can be opened in the open‐source software Paraview.
